# Safety signals of perfluorohexyloctane ophthalmic solution in patients with dry eye disease

**DOI:** 10.3389/fmed.2026.1832619

**Published:** 2026-05-28

**Authors:** Zhengtai Sun, Yuting Liu, Lijuan Que

**Affiliations:** Department of Ophthalmology, The First Affiliated Hospital of Soochow University, Suzhou, China

**Keywords:** adverse events, dry eye disease, FAERS, perfluorohexyloctane ophthalmic solution, pharmacovigilance

## Abstract

**Background:**

Dry eye disease (DED) is a common ocular surface disorder with limitations in conventional therapies. Perfluorohexyloctane ophthalmic solution, a novel formulation, has demonstrated short-term efficacy, but real-world safety data are insufficient. This study aimed to conduct a pharmacovigilance analysis using the FDA Adverse Event Reporting System (FAERS) database to provide evidence-based references for clinical rational drug use, risk management, and long-term DED treatment strategies.

**Methods:**

We conducted a retrospective pharmacovigilance study using FAERS data from the fourth quarter of 2023 through the fourth quarter of 2024, focusing on adverse events (AEs) associated with perfluorohexyloctane ophthalmic solution for DED. Four disproportionality analysis methods, the reporting odds ratio (ROR), the proportional reporting ratio (PRR), the Bayesian confidence propagation neural network (BCPNN), and the multi-item gamma Poisson shrinker (MGPS), were employed to identify potential safety signals.

**Results:**

A total of 92 AEs reports associated with perfluorohexyloctane ophthalmic solution for DED were identified. At the Preferred Term (PT) level, the top five reports by count included product delivery mechanism issue, eye irritation, inappropriate schedule of product administration, vision blurred, and ocular hyperaemia. The top five AEs meeting positive signal criteria were wrong dose, exposure via skin contact, patient dissatisfaction with treatment, product dose omission in error, and circumstance or information capable of leading to medication error. The median time-to-onset for perfluorohexyloctane-related AEs was 2 days.

**Conclusion:**

Overall, the available FAERS data do not raise major safety concerns for perfluorohexyloctane ophthalmic solution in the treatment of DED. The reported AEs appear to be mostly related to the instillation process, user experience with the delivery device, or the initial adaptation period, rather than directly attributable to the drug itself. Larger post-marketing studies are warranted to further characterize the safety profile of perfluorohexyloctane ophthalmic solution.

## 1 Introduction

Dry eye disease (DED) is a multifactorial disorder of the ocular surface characterized by loss of tear film homeostasis, often accompanied by ocular discomfort, visual disturbance, and tear film instability, potentially leading to ocular surface damage in severe cases (1). As one of the most prevalent ocular surface diseases globally, DED prevalence is increasing annually, significantly impairing patients’ quality of life, work productivity, and psychological well-being, thereby constituting a substantial public health burden (2). The global prevalence of DED ranges from 5.5 to 33.7%, while in China, it reaches 21.0 to 30.0%, with a trend toward a younger affected population (3). The pathophysiology of DED is complex; conventional treatments such as artificial tears and anti-inflammatory agents may alleviate some symptoms but have notable limitations in stabilizing the tear film lipid layer and achieving long-term disease control.

Perfluorohexyloctane ophthalmic solution, a water-free and preservative-free novel formulation approved by the U. S. Food and Drug Administration (FDA) for treating signs and symptoms of DED, has a unique mechanism of action. It forms a stable monolayer at the ocular surface, supplementing and stabilizing the endogenous tear film lipid layer, thereby effectively reducing tear evaporation (4). Multiple clinical studies have confirmed that this drug significantly improves both signs (e.g., tear film break-up time, corneal staining) and symptoms (e.g., Ocular Surface Disease Index score) in DED patients over short-term periods (weeks to months) (5, 6). A 52-week randomized controlled trial (KALAHARI study) further provided mid-term evidence, demonstrating good tolerability and sustained efficacy over 1 year of treatment (7).

Although randomized controlled trials are the gold standard for assessing drug efficacy and safety, their stringent inclusion/exclusion criteria, relatively homogenous study populations, and limited sample sizes may not fully capture the comprehensive safety profile of a drug in large-scale, long-term real-world use. Rare, delayed, or long-term adverse reactions that may emerge post-marketing necessitate continuous monitoring through pharmacovigilance systems. The FDA Adverse Event Reporting System (FAERS), a large public database based on spontaneous reports, provides valuable data for post-marketing safety research (8). Employing data mining techniques to analyze the FAERS database allows for the identification and quantification of statistical associations between drugs and potential AEs, thereby detecting safety signals, thereby effectively complementing the limitations of clinical trials. Currently, systematic pharmacovigilance studies based on large-scale real-world data regarding the long-term safety of perfluorohexyloctane ophthalmic solution for DED are lacking. Therefore, this study aims to conduct a comprehensive pharmacovigilance analysis of data related to this drug for DED using the FAERS database, to provide ophthalmic clinicians and regulatory agencies with real-world, long-term safety references. This will support clinical rational use and risk management while contributing population-level evidence from big data for formulating long-term DED management strategies.

## 2 Methods

### 2.1 Data source and collection

We conducted a retrospective pharmacovigilance assessment using the FAERS database, reviewing AE reports associated with perfluorohexyloctane ophthalmic solution from the fourth quarter of 2023 through the fourth quarter of 2024. The FAERS database aggregates information from various sources, including demographic and administrative details (DEMO), adverse reactions (REAC), patient outcomes (OUTC), drug-specific information (DRUG), therapy timelines (THER), reporter entity details (RPSR), and indications for use (INDI). This information was utilized to classify AEs based on individual patient drug exposure. We used the exact drug search terms and synonyms including Perfluorohexyloctane, Miebo, Siran, NovaTears, and Evotears.

Inclusion criteria:

(1) Confirmed diagnosis of DED.
(2) Patient age≥18 years.
(3) Patient received treatment with perfluorohexyloctane ophthalmic solution.
(4) Data recorded in the FAERS database.
(5) AE association criteria: Reported AEs must demonstrate a clear temporal relationship with perfluorohexyloctane ophthalmic solution use, i.e., the AE occurred during treatment or within a reasonable timeframe post-administration. Temporal association was assessed based on the THER and the REAC, requiring that the treatment start date precede the adverse reaction onset date.

Exclusion criteria:

(1) Duplicate reports: For duplicate reports of the same AE in the same patient, only the initial report data was retained. According to FDA recommendations, the PRIMARYID, CASEID, and FDA_DT fields are selected from the DEMO table to eliminate duplicate reports. The dataset is sorted by CASEID, FDA_DT, and PRIMARYID. For individual case safety reports (ICSRs) with the same CASEID, the report with the highest FDA_DT is retained. If multiple reports share the same CASEID and FDA_DT, the one with the highest PRIMARYID is selected (9).
(2) Significant missing data: Reports missing two or more key elements (e.g., patient age, sex, drug information, AE type) were excluded.
(3) Non-target drug association: Reports where the AE was clearly attributable to other drugs, diseases, or external factors without direct association with perfluorohexyloctane ophthalmic solution were excluded. Drugs coded as “concomitant medication,” “secondary suspect drug,” or “interaction drug” were excluded, as their causal relationship with the adverse events remains highly uncertain (10).
(4) Ambiguous information: Reports where key information, such as drug name or AE description, was unclear, preventing accurate determination of study eligibility, were excluded.

The detailed inclusion and exclusion process is shown in Supplementary Figure 1.

### 2.2 Signal detection data mining

The Proportional Reporting Ratio (PRR), the Reporting Odds Ratio (ROR), the Bayesian Confidence Propagation Neural Network (BCPNN), and the Multi-item Gamma Poisson Shrinker (MGPS) algorithms were applied to determine the statistical association between perfluorohexyloctane ophthalmic solution for DED and AEs. ROR, PRR, BCPNN, and MGPS are commonly used algorithms for disproportionality analysis, currently widely employed by the Medicines and Healthcare products Regulatory Agency (MHRA), the Netherlands Pharmacovigilance Center, the World Health Organization (WHO), and the FDA. ROR and PRR are frequentist (non-Bayesian) algorithms, with the advantage of correcting for bias in events with low reporting rates; PRR benefits from being less susceptible to underreporting of AEs. Non-Bayesian (frequency-based) methods offer simple computation and high sensitivity but carry a high risk of false positives when AE numbers are low (11). BCPNN and MGPS are Bayesian algorithms (12). BCPNN excels at integrating data from multiple sources and cross-validation (13); MGPS’s strength lies in detecting signals from rare events. Bayesian methods offer greater stability, account for uncertainty in small event sizes, reduce false alarm rates, and support higher-dimensional pattern recognition. However, they involve complex computations and exhibit relatively delayed signal detection. Therefore, this study employs a combined approach using multiple algorithms to leverage their respective strengths, broaden detection coverage, and validate results from multiple perspectives. This comprehensive methodology aims to identify more reliable safety signals (Supplementary Tables 1, 2). The final positive AE signal in this study was defined as satisfying the predefined thresholds of all four disproportionality algorithms simultaneously.

### 2.3 Data calculation

The AEs where perfluorohexyloctane ophthalmic solution was the primary suspect (PS) drug were extracted. The count data were described using case numbers and proportions. AE data from FAERS are coded using Preferred Terms from the Medical Dictionary for Regulatory Activities (MedDRA) maintained by the Council for International Organizations of Medical Sciences (CIOMS). AEs in this study are presented using the primary System Organ Class (SOC) and PT from MedDRA terminology.

### 2.4 Statistical analysis

All data processing and statistical analyses were performed using R software. Descriptive analysis summarized the clinical characteristics of patients with DED treated with perfluorohexyloctane ophthalmic solution. Time-to-onset (TTO) analysis was performed to investigate the onset time of AEs related to perfluorohexyloctane ophthalmic solution. Specifically, violin plots were constructed to visually characterize the distribution of TTO values, and Kaplan–Meier (KM) survival curves, designated as an exploratory analysis, were generated to depict the cumulative probability of AE onset over time for each SOC category due to the limited number of event reports at the PT level. Subgroup analyses were also conducted to mitigate the impact of demographic characteristics on the results.

## 3 Results

### 3.1 Baseline characteristics of AEs and population

A total of 92 AEs reports associated with perfluorohexyloctane ophthalmic solution for DED were identified (Supplementary Figure 1). The detailed temporal distribution was presented in Supplementary Figure 2. Table 1 presented the baseline data for these reports. Females (*n* = 81, 88.04%) accounted for a higher proportion of reporters compared to males (*n* = 11, 11.95%). Among the reporters, individuals aged 65–85 years (*n* = 59, 64.10%) constituted the largest group, followed by adults aged 18–64 years (*n* = 26, 28.30%), with those aged ≥86 years representing the smallest proportion (*n* = 7, 7.60%). The most frequently recorded outcome was missing (73 cases, 79.35%), followed by other (13 cases, 14.13%), hospitalization (3 cases, 3.26%), disability (2 cases, 2.17%), and death (1 case, 1.09%). The United States (*n* = 92, 100.00%) led in the number of adverse reaction reports. Reporters were predominantly consumers (*n* = 83, 90.22%), followed by physicians (*n* = 8, 8.70%) and health professionals (*n* = 1, 1.09%).

**Table 1:** Baseline characteristics.

Characteristics	Level	Overall
*n*		92
Age (year)		69.42 ± 11.08
Age group (%)	18 ~ 64	26(28.30)
	65 ~ 85	59(64.10)
	≥86	7(7.60)
Sex (%)	Male	11(11.95)
	Female	81(88.04)
Occupation (%)	CN	83(90.22)
	HP	1(1.09)
	MD	8(8.70)
Outcome (%)	DE	1(1.09)
	HO	3(3.26)
	DS	2(2.17)
	OT	13(14.13)
	Missing	73(79.35)
Death (%)	No	91(98.91)
	Yes	1(1.09)
Report Country (%)	USA	92(100.00)

CN, consumer; HP, health professional; MD, physician; DE, death; DS, disability; HO, hospitalization; OT, other.

### 3.2 Signal detection at the PT level

All AEs associated with perfluorohexyloctane ophthalmic solution for DED were systematically categorized by report count; detailed results were shown in Table 2. The top five PTs by report count included: product delivery mechanism issue, eye irritation, inappropriate schedule of product administration, vision blurred, and ocular hyperaemia. Among them, eye irritation, vision blurred, and ocular hyperaemia were drug-related AEs, while product delivery mechanism issue and inappropriate schedule of product administration were medication-related or device-related events.

**Table 2:** Risk signal of adverse reactions at the PT level (top 30 by report count).

PT	*N*	ROR (95%CI)	PRR (*χ*^2^)	EBGM (95%Cl)	IC (95%Cl)
Product delivery mechanism issue	20	31.12(19.08–50.77)	29.53(464.12)	24.97(16.58–37.6)	4.64(2.95–6.33)
Eye irritation	18	0.80(0.50–1.28)	0.81(0.88)	0.81(0.54–1.20)	−0.31(−1.98–1.36)
Inappropriate schedule of product administration	17	19.12(11.44–31.94)	18.30(249.42)	16.48(10.73–25.31)	4.04(2.35–5.73)
Vision blurred	16	1.23(0.75–2.04)	1.22(0.68)	1.22(0.80–1.86)	0.29(−1.38–1.96)
Ocular hyperaemia	12	1.34(0.75–2.39)	1.33(1.00)	1.33(0.82–2.15)	0.41(−1.26–2.08)
Product use issue	11	10.07(5.43–18.67)	9.80(82.03)	9.28(5.53–15.56)	3.21(1.53–4.90)
Product use complaint	10	15.43(8.00–29.78)	15.05(119.82)	13.81(7.97–23.93)	3.79(2.09–5.48)
Accidental exposure to product	10	5.74(3.03–10.88)	5.62(36.81)	5.46(3.20–9.31)	2.45(0.77–4.13)
Exposure via skin contact	10	99.51(44.86–220.73)	96.91(584.37)	60.02(30.82–116.9)	5.91(4.15–7.67)
Intentional product use issue	10	23.39(11.95–45.79)	22.80(181.97)	20.01(11.41–35.10)	4.32(2.62–6.03)
Eye pain	7	0.62(0.29–1.31)	0.63(1.59)	0.63(0.34–1.18)	−0.67(−2.34–1.01)
Product dose omission in error	7	85.04(33.74–214.36)	83.49(370.96)	54.62(25.20–118.38)	5.77(3.98–7.56)
Patient dissatisfaction with treatment	7	92.13(36.07–235.32)	90.45(391.21)	57.49(26.23–126.00)	5.85(4.05–7.64)
Wrong dose	7	138.21(49.86–383.08)	135.67(499.19)	72.82(31.03–170.91)	6.19(4.37–8.01)
Visual impairment	7	2.19(1.03–4.66)	2.17(4.40)	2.15(1.15–4.05)	1.11(−0.57–2.78)
Product packaging quantity issue	6	9.53(4.16–21.87)	9.40(42.52)	8.92(4.45–17.86)	3.16(1.46–4.85)
Headache	5	1.01(0.42–2.44)	1.01(0.00)	1.01(0.48–2.11)	0.01(−1.66–1.69)
Foreign body sensation in eyes	5	1.25(0.52–3.03)	1.25(0.24)	1.24(0.59–2.61)	0.32(−1.36–1.99)
Lacrimation increased	5	1.02(0.42–2.47)	1.02(0.00)	1.02(0.49–2.14)	0.03(−1.65–1.70)
Eye pruritus	5	1.00(0.41–2.42)	1.00(0.00)	1.00(0.48–2.10)	0.00(−1.68–1.68)
Therapy interrupted	5	10.60(4.26–26.38)	10.48(40.20)	9.88(4.61–21.17)	3.30(1.60–5.01)
Circumstance or information capable of leading to medication error	5	60.42(21.43–170.33)	59.64(208.25)	43.35(18.21–103.18)	5.44(3.64–7.24)
Dry eye	4	0.62(0.23–1.66)	0.62(0.93)	0.62(0.27–1.43)	−0.68(−2.35–0.99)
Photophobia	4	2.21(0.82–5.97)	2.20(2.59)	2.18(0.95–5.01)	1.13(−0.55–2.81)
Product complaint	4	15.28(5.45–42.87)	15.13(48.12)	13.87(5.85–32.89)	3.79(2.07–5.52)
Cerebrovascular accident	3	6.79(2.13–21.66)	6.74(14.08)	6.50(2.46–17.17)	2.70(0.99–4.41)
Drug ineffective	3	0.33(0.11–1.02)	0.33(4.09)	0.33(0.13–0.87)	−1.58(−3.25–0.10)
Blindness transient	3	10.41(3.22–33.65)	10.34(23.74)	9.75(3.65–26.03)	3.29(1.56–5.01)
Eye swelling	3	1.05(0.34–3.29)	1.05(0.01)	1.05(0.41–2.73)	0.07(−1.60–1.75)

The preferred term “Product distribution issue” (*n* = 4) is presented descriptively only in this note. Its disproportionality analysis results are as follows: ROR = Inf (NaN-Inf), PRR = Inf (620.25), EBGM = 156.05 (0-Inf), and IC = 7.29 (5.17–9.40). Infinite ROR/PRR values arise because this term was not reported with other drugs in the database, leading to mathematically undefined estimates. These values are not included in the main table and should be interpreted with caution. PT, preferred term; ROR, reporting odds ratio; CI, confidence interval; PRR, proportional reporting ratio; *χ*^2^, chi-squared; EBGM, empirical Bayesian geometric mean; IC, information component.

The top five AEs meeting positive signal criteria included: wrong dose, exposure via skin contact, patient dissatisfaction with treatment, product dose omission in error, and circumstance or information capable of leading to medication error. These first five positive signals were all medication-related or device-related events. Among the positive signals, only cerebrovascular accident and transient blindness were drug-related AEs.

### 3.3 Time-to-onset analysis at SOC level

**Figure 1:**
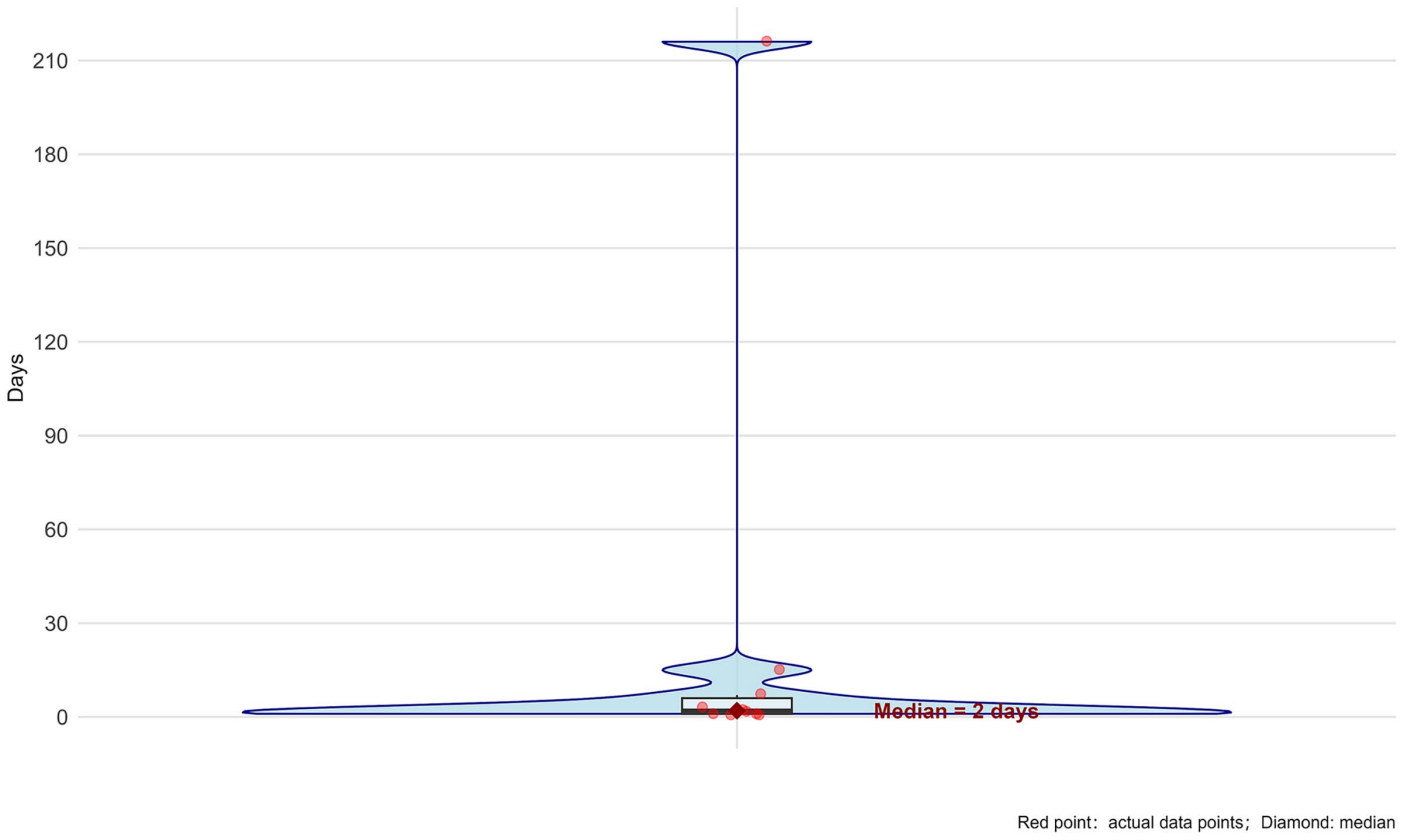
Violin plot of onset time of adverse events in perfluorohexyloctane.

**Figure 2:**
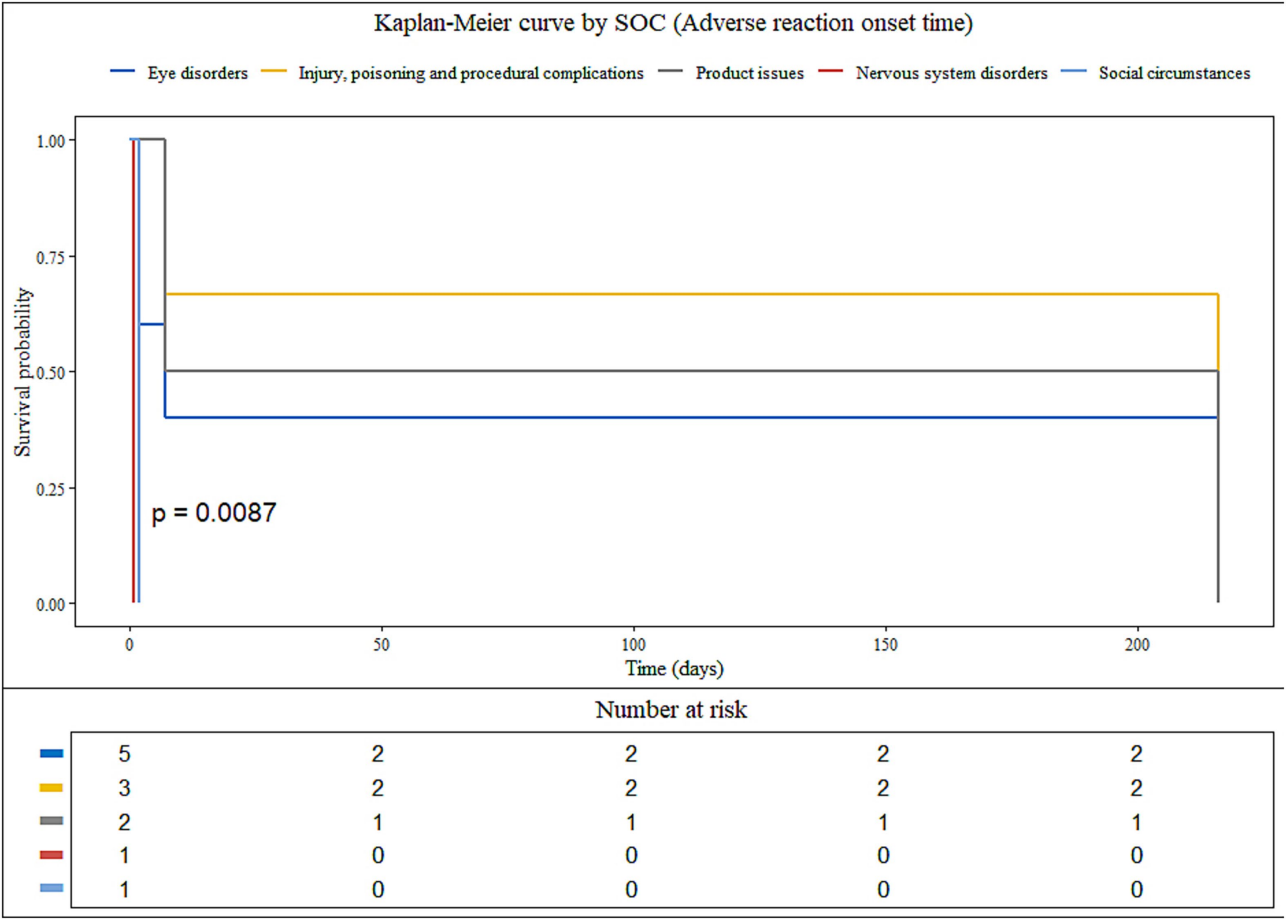
Kaplan–Meier curve of time to onset of adverse events for perfluorohexyloctane at the SOC level. SOC, system organ class.

The TTO analysis revealed a median onset time of 2 days for AEs related to perfluorohexyloctane ophthalmic solution (Figure 1). Given the small number of event reports at the PT level, the KM survival curve analysis in this study was ultimately performed at the SOC level. In an exploratory Kaplan–Meier analysis, the curves showed a difference in the distribution of AE onset times across different SOCs (Figure 2).

### 3.4 Subgroup analysis

#### 3.4.1 Sex subgroup

At the PT level, the top five PTs by report count in the male subgroup included: drug ineffective, product dose omission in error, product packaging quantity issue, product use complaint, and product delivery mechanism issue (Supplementary Table 3). All five of the above were medication-related or device-related events. The top five PTs by report count in the female subgroup included: product delivery mechanism issue, eye irritation, Inappropriate schedule of product administration, vision blurred, and ocular hyperaemia (Supplementary Table 4). Among them, eye irritation, vision blurred, and ocular hyperaemia were drug-related AEs, while product delivery mechanism issue and inappropriate schedule of product administration were medication-related or device-related events. No positive signal was identified in the male subgroup. In the female subgroup, the top five AEs meeting positive signal criteria included: wrong dose, product design issue, exposure via skin contact, patient dissatisfaction with treatment, and product dose omission in error. These first five positive signals were all medication-related or device-related events. Among the positive signals, only cerebrovascular accident and transient blindness were drug-related AEs.

#### 3.4.2 Age subgroup

At the PT level, the top five PTs by report count in the 18–64 years subgroup included: Inappropriate schedule of product administration, vision blurred, ocular hyperaemia, eye irritation, and product delivery mechanism issue (Supplementary Table 5). Among them, eye irritation, vision blurred, and ocular hyperaemia were drug-related AEs, while product delivery mechanism issue and inappropriate schedule of product administration were medication-related or device-related events. The top five PTs by report count in the ≥65 years subgroup mainly included: product delivery mechanism issue, eye irritation, vision blurred, inappropriate schedule of product administration, and product use issue (Supplementary Table 6). Among them, eye irritation and vision blurred are drug-related AEs, while product delivery mechanism issue, inappropriate schedule of product administration, and product use issue are medication-related or device-related events. In the 18–64 years subgroup, the top five AEs meeting positive signal criteria were: exposure via skin contact, product delivery mechanism issue, inappropriate schedule of product administration, product use complaint, and accidental exposure to product. These first five positive signals were all medication-related or device-related events. There were no drug-related AEs among the positive signals. In the ≥65 years subgroup, the top five AEs meeting positive signal criteria were: wrong dose, product dose omission in error, exposure via skin contact, patient dissatisfaction with treatment, and circumstance or information capable of leading to medication error. These first five positive signals are all medication-related or device-related events. Among the positive signals, only cerebrovascular accident was a drug-related AE.

## 4 Discussion

This study represents the first systematic pharmacovigilance analysis of AEs associated with perfluorohexyloctane ophthalmic solution for DED based on large-scale real-world data from the FAERS. By mining 92 case reports, we identified potential safety signals related to this drug and conducted in-depth analyses of demographic characteristics, time-to-onset, and subgroup differences. These findings provide important supplementary information regarding the medication use-related issues and the importance of early patient education for this novel anti-evaporative dry eye therapy in real-world clinical settings.

The relatively limited number of AE reports identified (*n* = 92) partly reflects the level of exposure to perfluorohexyloctane as a recently marketed drug and may also suggest its generally favorable safety profile, consistent with findings from pivotal clinical trials and extension studies (7). In the GOBI (14) and MOJAVE (15) Phase III trials, the KALAHARI extension study (7) and other research (16), perfluorohexyloctane demonstrated a good safety profile and sustained efficacy, with the most common AE being mild, transient blurred vision (17). A recent meta-analysis also confirmed the efficacy of perfluorohexyloctane in improving corneal staining and dry eye symptoms, with a safety profile comparable to that of other DED treatments (18, 19).

At the PT level, the most frequently reported signals included product delivery mechanism issue, eye irritation, vision blurred, and ocular hyperaemia. Notably, signals meeting positive criteria across multiple disproportionality analysis algorithms, such as wrong dose, exposure via skin contact, and patient dissatisfaction with treatment were primarily related to the use of the delivery device, patient handling, or medication adherence, rather than direct drug AEs. This suggests that some reported adverse experiences may be associated with patient unfamiliarity when first using this novel, water-free, low-surface-tension eye drop. As a semifluorinated alkane, perfluorohexyloctane possesses both lipophilic and hydrophobic amphiphilic properties, enabling it to rapidly form a stable monolayer at the air-liquid interface of the tear film, thereby supplementing the deficient lipid layer and inhibiting tear fluid evaporation (5, 18, 20). Preclinical studies have shown that perfluorohexyloctane spreads extremely rapidly over the ocular surface, and this rapid spreading behavior facilitates the formation of a long-lasting anti-evaporative barrier (21). The rapid spreading of this drug after instillation can instantly alter the refractive properties of the tear film and the smoothness of the air-tear interface, inducing optical interfacial effects that manifest as transient blurred vision (22). Furthermore, the monolayer formed on the tear film surface may cause temporary changes in tear film dynamics, which can also lead to mild eye irritation and conjunctival hyperemia to some extent. The above AEs are largely predictable based on the compound’s physicochemical properties and the behavior of eye drop instillation, and may partially reflect the physiological response of the ocular surface to this novel anhydrous, low-surface-tension ophthalmic formulation.

In this study, only two drug-related AEs were identified: cerebrovascular accident and transient blindness. The signal for cerebrovascular accident was particularly prominent in the overall population, the female subgroup, and the ≥65 years subgroup. Perfluorohexyloctane is a topically applied ophthalmic formulation with extremely low systemic bioavailability, and current pharmacological studies and clinical trials have not found it to possess direct systemic vasoactive or prothrombotic effects (7, 23). Therefore, the observed signal is highly likely to be confounded. The reported population is predominantly elderly females, a group that itself has a high prevalence of risk factors for cerebrovascular diseases such as hypertension and atrial fibrillation (24, 25). Temporal associations in spontaneous reports do not prove causality; these cerebrovascular events are likely related to the patients’ underlying pre-existing conditions rather than directly caused by the drug. Nevertheless, this signal suggests that when providing routine medical monitoring in elderly patients using this drug, clinicians should remain attentive to their overall cardiovascular risk. Transient blindness is closely associated with the drug’s known and most common adverse reaction, blurred vision. In a spontaneous reporting system, patients may subjectively describe a sudden, severe episode of blurred vision as “transient blindness.” Additionally, transient blindness may be related to patients’ underlying ocular conditions rather than the direct toxic effects of the drug. This underscores the importance of adequately informing patients during the early stages of treatment to manage their expectations and reduce unnecessary anxiety.

The TTO analysis showed a median onset time of only 2 days for Perfluorohexyloctane-related AEs, and Kaplan–Meier curves presented trends of differences in the distribution of onset times across different SOCs. The vast majority of AEs were reported very early in treatment (within days to weeks), suggesting that many adverse experiences are associated with the adaptation process during the initial treatment phase. This pattern of early onset supported the notion that these AEs are predominantly transient and related to the drug’s local effects or its method of use. This finding carries significant clinical implications: healthcare providers should provide thorough instructions and expectation management when initiating perfluorohexyloctane therapy, informing patients about potential transient visual changes or ocular discomfort to enhance treatment adherence and satisfaction (26).

The significantly higher proportion of female reporters (88.0%) and the predominance of patients aged over 65 years (64.1%) align with the known higher prevalence of DED in women and the elderly (27), and may also reflect a greater propensity in these groups to seek treatment or report health issues. Subgroup analysis revealed that positive signals were primarily concentrated in the female and ≥65 years age groups. This discrepancy may stem from insufficient statistical power due to imbalanced sample sizes, or could suggest genuine differences in how different populations perceive and react to the drug or its administration. For instance, elderly patients might encounter more challenges in handling the eye drop due to reduced manual dexterity or visual acuity, leading to more use-related reports.

This study has several limitations. First, the FAERS spontaneous reporting system has issues such as reporting bias (most AE reports are submitted by non-professionals, leading to insufficient clinical quality and frequent use of non-technical terms), over-reporting by users (especially for recently marketed drugs), under-reporting, and incomplete information (e.g., concomitant medications, disease duration, etc.). Therefore, it is not possible to calculate the true incidence of AEs or directly infer causality. Second, the sample size of this study is relatively small, which may be insufficient to detect rare but serious AEs. Moreover, due to the limited sample size, the survival analysis lacks adequate statistical power, and the relevant conclusions need to be further validated by real-world studies with larger sample sizes. Third, the FAERS data used in this study are all from the United States (accounting for 100%), which may limit the generalizability of the findings to other racial and geographical populations. Fourth, although subgroup analyses were performed, the unbalanced sample sizes across subgroups may affect the stability of the results. In particular, the high proportion of missing weight data precluded effective weight-stratified analysis. Future studies should expand the sample size, improve data collection, and further explore the impact of weight on the risk of AEs. Fifth, the absence of outcome data prevents risk-weighted assessment based on severity, which is a major factor limiting the clinical utility of this study. Future large-scale, long-term prospective observational studies are needed to more precisely quantify the incidence of AEs associated with perfluorohexyloctane and to confirm its long-term safety. Additionally, optimizing the ergonomic design of the drug delivery device and providing clearer instructions should be considered.

## 5 Conclusion

Overall, the available FAERS data do not raise major safety concerns for perfluorohexyloctane ophthalmic solution in the treatment of DED. The reported AEs appear to be mostly related to the instillation process, user experience with the delivery device, or the initial adaptation period, rather than directly attributable to the drug itself. Larger post-marketing studies are warranted to further characterize the safety profile of perfluorohexyloctane ophthalmic solution.

## Data Availability

The original contributions presented in the study are included in the article/Supplementary material, further inquiries can be directed to the corresponding author.

## Supplementary material

SUPPLEMENTARY FIGURE 1 Flow chart.

SUPPLEMENTARY FIGURE 2 Detailed time distribution.
