# Supplementary figures and images for: Safety signals of perfluorohexyloctane ophthalmic solution in patients with dry eye disease
Source: Front Med (Lausanne). 2026 May 28;13:1832619. doi: 10.3389/fmed.2026.1832619 (PMC13262190; doi:10.3389/fmed.2026.1832619)

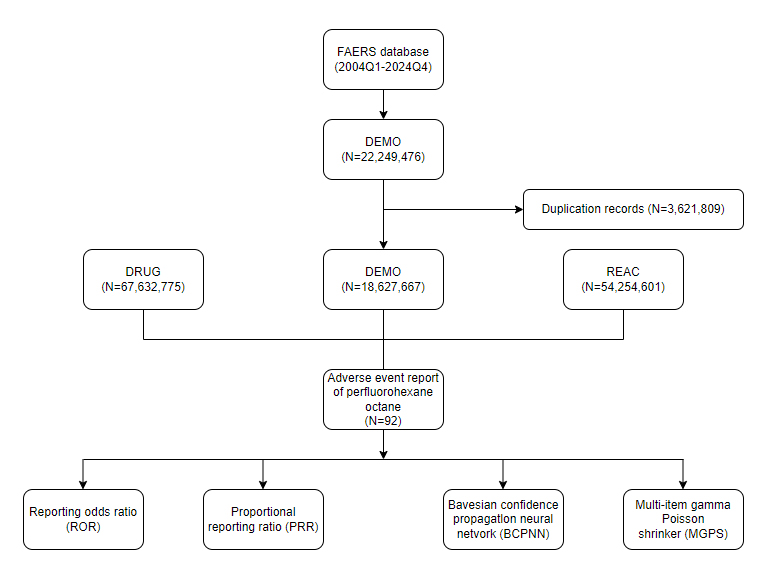

Supplement: SUPPLEMENTARY FIGURE 1 — Flow chart. [file Image_1.JPEG]

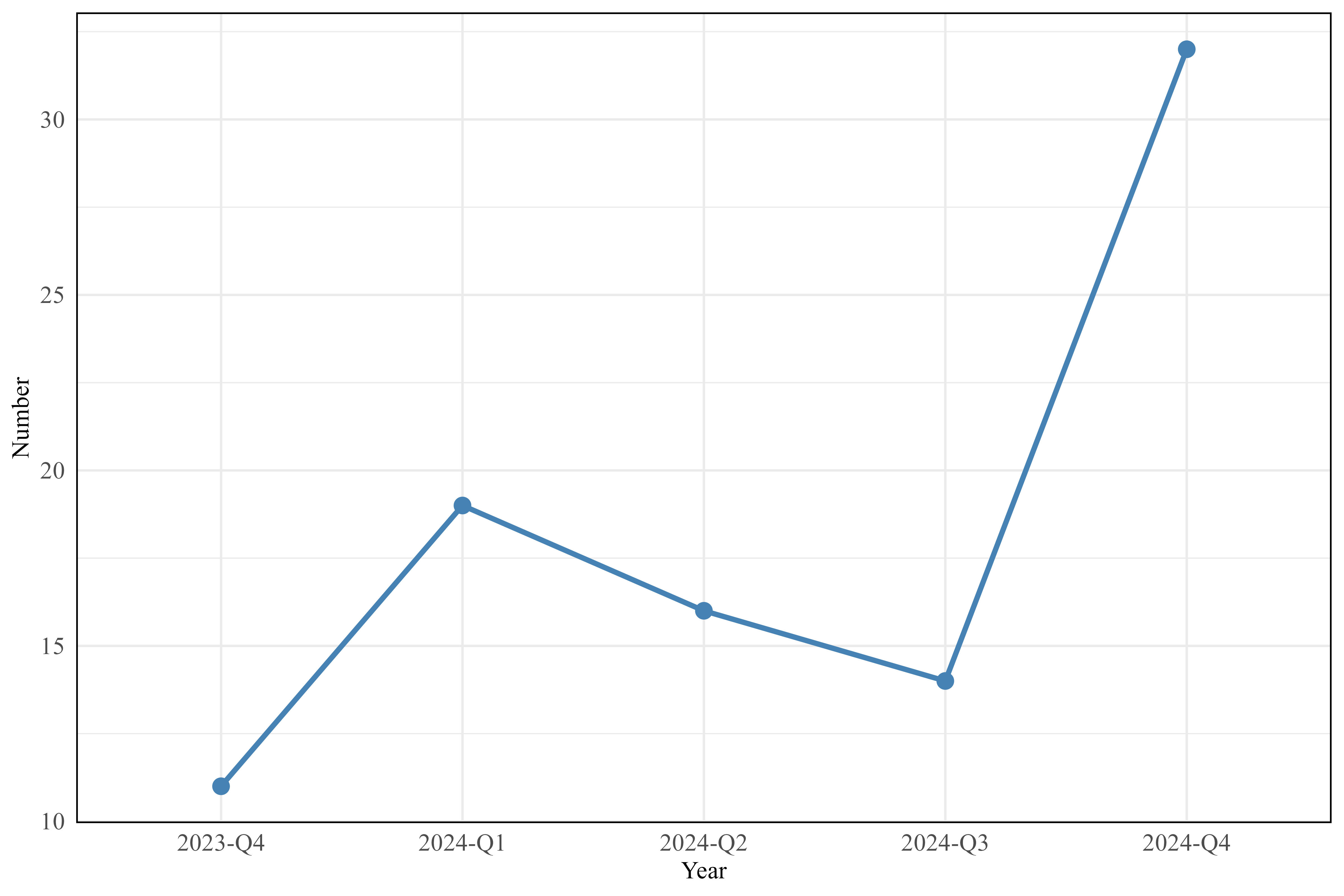

Supplement: SUPPLEMENTARY FIGURE 2 — Detailed time distribution. [file Image_2.JPEG]
