# Supplementary material for: Safety signals of perfluorohexyloctane ophthalmic solution in patients with dry eye disease
Source: Front Med (Lausanne). 2026 May 28;13:1832619. doi: 10.3389/fmed.2026.1832619 (PMC13262190; doi:10.3389/fmed.2026.1832619)
Supplement: Supplementary file 3 [file Table_1.DOCX]

Supplementary Table 1 Fourfold table of disproportionality analyses

| Medicine | Target adverse events reported | Other adverse events reported | Summation |
| --- | --- | --- | --- |
| Target drugs | a | b | a+b |
| Other drugs | c | d | c+d |
| Summation | a+c | b+d | N=a+b+c+d |
