# Supplementary material for: Safety signals of perfluorohexyloctane ophthalmic solution in patients with dry eye disease
Source: Front Med (Lausanne). 2026 May 28;13:1832619. doi: 10.3389/fmed.2026.1832619 (PMC13262190; doi:10.3389/fmed.2026.1832619)
Supplement: Supplementary file 4 [file Table_2.DOCX]

Supplementary Table 2. Four algorithms used for signal detection

| Algorithm | Equation | Criteria |
| --- | --- | --- |
| ROR | ROR = ad/bc | Lower limit of 95% CI > 1, N ≥ 3 |
|  | 95%CI = e^ln(ROR)±1.96(1/a+1/b+1/c+1/d)^0.5^ |  |
| PRR | PRR = [a(c+d)]/[c(a+b)] | PRR ≥ 2, *χ^2^*≥ 4, N ≥ 3 |
|  | *χ^2^*=[(ad-bc)^2](a+b+c+d)/[(a+b)(c+d)(a+c)(b+d)] |  |
| BCPNN | IC = log_2_a(a+b+c+d)/[(a+c)(a+b)] | IC025 > 0 |
|  | 95%*CI* = E(IC) ± 2[V(IC)]^0.5 |  |
| MGPS | EBGM = a(a+b+c+d)/[(a+c)(a+b)] | EBGM05 > 2 |
|  | 95%*CI* = e^ln(EBGM)±1.96(1/a+1/b+1/c+1/d)^0.5^ |  |

ROR: reporting odds ratio; PRR: proportional reported ratio; BCPNN: Bayesian confidence propagation neural network; MGPS: multiple Gamma Poisson Shrinker; EBGM: empirical Bayesian geometric mean; CI: confidence interval; a: the number of reports containing both target drug and target adverse drug reaction; b: the number of reports containing the target adverse drug reaction with other medications (except the target drug); c: the number of reports containing the target drug with other adverse drug reactions (except the target events); d: the number of all reports.
