# Supplementary material for: Safety signals of perfluorohexyloctane ophthalmic solution in patients with dry eye disease
Source: Front Med (Lausanne). 2026 May 28;13:1832619. doi: 10.3389/fmed.2026.1832619 (PMC13262190; doi:10.3389/fmed.2026.1832619)
Supplement: Supplementary file 5 [file Table_3.DOCX]

Supplementary Table 3 Risk signal of adverse reactions at the PT levels stratified in the male population

| PT | N | ROR(95%Cl) | PRR($\chi^{2}$) | EBGM(95%Cl) | IC(95%Cl) |
| --- | --- | --- | --- | --- | --- |
| Drug ineffective | 2 | 1.63(0.39-6.73) | 1.60(0.46) | 1.60(0.49-5.24) | 0.68(-1.04-2.40) |
| Product dose omission in error | 2 | 134.57(22.01-822.86) | 129.44(153.01) | 78.06(17.16-355.15) | 6.29(4.21-8.36) |
| Product packaging quantity issue | 2 | 23.72(5.34-105.36) | 22.84(37.44) | 20.54(5.90-71.54) | 4.36(2.53-6.19) |
| Product use complaint | 2 | 23.72(5.34-105.36) | 22.84(37.44) | 20.54(5.90-71.54) | 4.36(2.53-6.19) |
| Product delivery mechanism issue | 2 | 28.81(6.38-130.06) | 27.74(45.17) | 24.39(6.91-86.12) | 4.61(2.76-6.46) |
| Vision blurred | 2 | 1.04(0.25-4.30) | 1.04(0.00) | 1.04(0.32-3.41) | 0.06(-1.66-1.78) |
| Ocular hyperaemia | 2 | 2.14(0.52-8.87) | 2.1(1.16) | 2.09(0.64-6.85) | 1.06(-0.66-2.79) |
| Patient dissatisfaction with treatment | 2 | 100.92(18.07-563.59) | 97.08(126.86) | 65.05(15.42-274.34) | 6.02(3.99-8.05) |
| Product after taste | 1 | 197.94(12.21-3208.15) | 194.15(96.10) | 97.58(9.49-1003.60) | 6.61(4.22-9.00) |
| Nausea | 1 | 3.58(0.49-26.37) | 3.53(1.79) | 3.48(0.66-18.53) | 1.80(0.05-3.56) |
| Blood pressure increased | 1 | 10.40(1.37-79.15) | 10.22(7.92) | 9.76(1.79-53.32) | 3.29(1.46-5.11) |
| Tachycardia | 1 | 98.96(8.83-1108.70) | 97.08(63.41) | 65.05(8.61-491.23) | 6.02(3.78-8.26) |
| Product odour abnormal | 1 | 98.96(8.83-1108.70) | 97.08(63.41) | 65.05(8.61-491.23) | 6.02(3.78-8.26) |
| Asthenopia | 1 | 15.21(1.95-118.43) | 14.93(12.09) | 13.94(2.50-77.64) | 3.80(1.93-5.67) |
| Photosensitivity reaction | 1 | 24.73(3.04-201.31) | 24.27(19.85) | 21.68(3.75-125.36) | 4.44(2.50-6.38) |
| Accidental exposure to product | 1 | 4.58(0.62-33.93) | 4.52(2.69) | 4.44(0.83-23.68) | 2.15(0.38-3.91) |
| Exposure via skin contact | 1 | 65.97(6.75-644.86) | 64.72(47.07) | 48.79(7.24-328.70) | 5.61(3.46-7.75) |
| Dysphonia | 1 | 17.98(2.28-141.83) | 17.65(14.42) | 16.26(2.89-91.58) | 4.02(2.13-5.91) |
| Lacrimation increased | 1 | 1.67(0.23-12.20) | 1.66(0.26) | 1.65(0.31-8.72) | 0.73(-1.01-2.46) |
| Inappropriate schedule of product administration | 1 | 7.90(1.05-59.41) | 7.77(5.68) | 7.51(1.39-40.61) | 2.91(1.11-4.71) |
| Periorbital swelling | 1 | 24.73(3.04-201.31) | 24.27(19.85) | 21.68(3.75-125.36) | 4.44(2.50-6.38) |
| Foreign body sensation in eyes | 1 | 2.49(0.34-18.21) | 2.46(0.86) | 2.44(0.46-12.91) | 1.29(-0.46-3.03) |
| Diplopia | 1 | 14.12(1.82-109.4) | 13.87(11.16) | 13.01(2.35-72.16) | 3.70(1.84-5.56) |
| Eye swelling | 1 | 5.19(0.70-38.52) | 5.11(3.23) | 5.00(0.94-26.78) | 2.32(0.55-4.09) |
| Erythema | 1 | 5.06(0.68-37.51) | 4.98(3.11) | 4.88(0.91-26.09) | 2.29(0.52-4.06) |
| Eye pain | 1 | 0.71(0.10-5.18) | 0.72(0.11) | 0.72(0.14-3.78) | -0.47(-2.19-1.25) |
| Headache | 1 | 1.76(0.24-12.88) | 1.75(0.32) | 1.74(0.33-9.20) | 0.80(-0.93-2.53) |
| Conjunctival hyperaemia | 1 | 65.97(6.75-644.86) | 64.72(47.07) | 48.79(7.24-328.7) | 5.61(3.46-7.75) |
| Rhinorrhoea | 1 | 8.98(1.19-67.88) | 8.83(6.65) | 8.48(1.56-46.10) | 3.08(1.27-4.90) |

Note: For "Localised oedema" (n=1), ROR = Inf (NaN-Inf), PRR = Inf (194.17), EBGM = 195.15 (0-Inf), IC = 7.61 (4.95-10.27). This entry is presented descriptively only in this note, not included in the main table. Infinite values occur because this preferred term was not reported with other drugs, leading to mathematically undefined estimates. PT: preferred term; ROR: reporting odds ratio; CI: confidence interval; PRR: proportional reporting ratio; χ2: chi-squared; EBGM: empirical Bayesian geometric mean; IC: information component.
