# Supplementary material for: Safety signals of perfluorohexyloctane ophthalmic solution in patients with dry eye disease
Source: Front Med (Lausanne). 2026 May 28;13:1832619. doi: 10.3389/fmed.2026.1832619 (PMC13262190; doi:10.3389/fmed.2026.1832619)
Supplement: Supplementary file 6 [file Table_4.DOCX]

Supplementary Table 4 Risk signal of adverse reactions at the PT levels in the female population

| PT | N | ROR(95%Cl) | PRR($\chi^{2}$) | EBGM(95%Cl) | IC(95%Cl) |
| --- | --- | --- | --- | --- | --- |
| Product delivery mechanism issue | 18 | 31.10(18.53-52.19) | 29.44(413.78) | 24.74(16.04-38.16) | 4.63(2.93-6.32) |
| Eye irritation | 17 | 0.87(0.53-1.42) | 0.88(0.32) | 0.88(0.58-1.32) | -0.19(-1.86-1.48) |
| Inappropriate schedule of product administration | 16 | 20.99(12.31-35.79) | 20.01(255.44) | 17.76(11.36-27.75) | 4.15(2.46-5.84) |
| Vision blurred | 14 | 1.27(0.74-2.18) | 1.26(0.77) | 1.26(0.80-1.97) | 0.33(-1.34-2.01) |
| Ocular hyperaemia | 10 | 1.23(0.66-2.32) | 1.23(0.43) | 1.23(0.72-2.08) | 0.29(-1.38-1.97) |
| Product use issue | 10 | 11.18(5.83-21.44) | 10.86(83.72) | 10.19(5.91-17.58) | 3.35(1.66-5.04) |
| Accidental exposure to product | 9 | 5.88(3.00-11.55) | 5.75(34.16) | 5.57(3.17-9.80) | 2.48(0.79-4.16) |
| Intentional product use issue | 9 | 23.72(11.65-48.29) | 23.10(164.92) | 20.13(11.10-36.48) | 4.33(2.62-6.04) |
| Exposure via skin contact | 9 | 105.93(44.96-249.58) | 103.04(537.64) | 61.30(29.92-125.57) | 5.94(4.16-7.72) |
| Product use complaint | 8 | 14.17(6.81-29.48) | 13.85(87.39) | 12.75(6.91-23.54) | 3.67(1.97-5.37) |
| Wrong dose | 7 | 152.07(53.03-436.05) | 148.83(514.09) | 74.92(31.03-180.88) | 6.23(4.40-8.06) |
| Visual impairment | 7 | 2.56(1.20-5.44) | 2.52(6.38) | 2.50(1.33-4.70) | 1.32(-0.36-3.00) |
| Eye pain | 6 | 0.60(0.27-1.36) | 0.61(1.51) | 0.61(0.31-1.21) | -0.70(-2.38-0.97) |
| Product dose omission in error | 5 | 75.56(25.68-222.3) | 74.42(241.51) | 49.94(20.25-123.21) | 5.64(3.82-7.46) |
| Patient dissatisfaction with treatment | 5 | 94.45(30.73-290.27) | 93.02(280.17) | 57.63(22.52-147.44) | 5.85(4.01-7.69) |
| Therapy interrupted | 5 | 12.37(4.94-31.00) | 12.20(47.58) | 11.35(5.26-24.48) | 3.50(1.79-5.21) |
| Headache | 4 | 0.90(0.34-2.43) | 0.9(0.04) | 0.91(0.40-2.07) | -0.14(-1.82-1.53) |
| Dry eye | 4 | 0.70(0.26-1.89) | 0.71(0.50) | 0.71(0.31-1.62) | -0.50(-2.18-1.18) |
| Foreign body sensation in eyes | 4 | 1.10(0.41-2.95) | 1.10(0.03) | 1.10(0.48-2.51) | 0.13(-1.55-1.81) |
| Lacrimation increased | 4 | 0.92(0.34-2.49) | 0.93(0.02) | 0.93(0.40-2.12) | -0.11(-1.79-1.57) |
| Eye pruritus | 4 | 0.85(0.32-2.30) | 0.86(0.10) | 0.86(0.37-1.96) | -0.22(-1.90-1.45) |
| Product packaging quantity issue | 4 | 7.34(2.67-20.14) | 7.26(20.62) | 6.97(2.99-16.22) | 2.80(1.10-4.50) |
| Circumstance or information capable of leading to medication error | 4 | 50.21(16.11-156.52) | 49.61(142.94) | 37.46(14.47-96.98) | 5.23(3.41-7.04) |
| Cerebrovascular accident | 3 | 10.98(3.38-35.64) | 10.89(25.13) | 10.22(3.81-27.36) | 3.35(1.63-5.08) |
| Blindness transient | 3 | 12.87(3.94-42.05) | 12.76(29.96) | 11.83(4.39-31.86) | 3.56(1.83-5.30) |
| Product dose omission issue | 3 | 3.98(1.26-12.59) | 3.95(6.46) | 3.88(1.48-10.16) | 1.95(0.26-3.65) |
| Product design issue | 3 | 112.65(25.11-505.35) | 111.63(187.97) | 64.21(18.29-225.46) | 6.00(4.05-7.96) |
| Photophobia | 3 | 1.87(0.60-5.86) | 1.86(1.18) | 1.85(0.71-4.82) | 0.89(-0.80-2.57) |
| Product residue present | 3 | 32.18(9.20-112.51) | 31.89(73.96) | 26.44(9.28-75.36) | 4.72(2.92-6.53) |
| Therapeutic product effect incomplete | 3 | 10.72(3.31-34.76) | 10.63(24.45) | 9.99(3.73-26.73) | 3.32(1.59-5.05) |

PT: preferred term; ROR: reporting odds ratio; CI: confidence interval; PRR: proportional reporting ratio; χ2: chi-squared; EBGM: empirical Bayesian geometric mean; IC: information component.
