# Supplementary material for: Safety signals of perfluorohexyloctane ophthalmic solution in patients with dry eye disease
Source: Front Med (Lausanne). 2026 May 28;13:1832619. doi: 10.3389/fmed.2026.1832619 (PMC13262190; doi:10.3389/fmed.2026.1832619)
Supplement: Supplementary file 7 [file Table_5.DOCX]

Supplementary Table 5 Risk signal of adverse reactions at the PT levels in people aged 18 to 64

| PT | N | ROR(95%Cl) | PRR($\chi^{2}$) | EBGM(95%Cl) | IC(95%Cl) |
| --- | --- | --- | --- | --- | --- |
| Inappropriate schedule of product administration | 6 | 38.16(15.85-91.9) | 35.84(178.66) | 31.57(15.13-65.87) | 4.98(3.24-6.72) |
| Vision blurred | 5 | 1.83(0.74-4.53) | 1.79(1.79) | 1.79(0.84-3.80) | 0.84(-0.86-2.53) |
| Ocular hyperaemia | 5 | 2.16(0.87-5.33) | 2.10(2.92) | 2.09(0.98-4.45) | 1.06(-0.63-2.76) |
| Eye irritation | 5 | 1.00(0.41-2.46) | 1.00(0.00) | 1.00(0.47-2.13) | 0.00(-1.69-1.69) |
| Product delivery mechanism issue | 4 | 66.96(21.96-204.11) | 64.21(199.28) | 51.57(20.29-131.04) | 5.69(3.88-7.49) |
| Eye pruritus | 3 | 2.48(0.78-7.89) | 2.44(2.55) | 2.42(0.92-6.37) | 1.28(-0.42-2.98) |
| Eye pain | 3 | 1.17(0.37-3.71) | 1.17(0.07) | 1.16(0.44-3.06) | 0.22(-1.47-1.92) |
| Product use complaint | 3 | 25.62(7.70-85.29) | 24.85(62.71) | 22.75(8.32-62.22) | 4.51(2.75-6.27) |
| Accidental exposure to product | 3 | 10.57(3.28-34.13) | 10.27(24.22) | 9.92(3.72-26.44) | 3.31(1.59-5.03) |
| Exposure via skin contact | 3 | 159.04(37.46- 675.15) | 154.10(285.29) | 96.69(28.84-324.16) | 6.60(4.65-8.54) |
| Headache | 2 | 1.43(0.35-5.83) | 1.42(0.25) | 1.42(0.44-4.60) | 0.51(-1.19-2.20) |
| Product after taste | 1 | 37.07(4.52-304.20) | 36.69(30.39) | 32.23(5.54-187.58) | 5.01(3.06-6.96) |
| Nausea | 1 | 1.22(0.17-8.79) | 1.22(0.04) | 1.22(0.23-6.35) | 0.28(-1.42-1.99) |
| Blood pressure increased | 1 | 7.00(0.95-51.57) | 6.94(4.96) | 6.79(1.28-36.06) | 2.76(1.01-4.52) |
| Tachycardia | 1 | 23.58(3.01-184.49) | 23.35(19.62) | 21.49(3.84-120.13) | 4.43(2.55-6.30) |
| Product odour abnormal | 1 | 37.07(4.52-304.20) | 36.69(30.39) | 32.23(5.54-187.58) | 5.01(3.06-6.96) |
| Eye haemorrhage | 1 | 8.94(1.21-66.29) | 8.86(6.75) | 8.59(1.61-45.95) | 3.10(1.33-4.87) |
| Liquid product physical issue | 1 | 19.95(2.58-154.06) | 19.76(16.54) | 18.42(3.33-101.85) | 4.20(2.35-6.05) |
| Drug ineffective | 1 | 0.47(0.07-3.40) | 0.48(0.58) | 0.48(0.09-2.50) | -1.06(-2.76-0.63) |
| Asthenopia | 1 | 4.46(0.61-32.56) | 4.43(2.62) | 4.37(0.83-23.04) | 2.13(0.40-3.86) |
| Lacrimation increased | 1 | 0.93(0.13-6.67) | 0.93(0.01) | 0.93(0.18-4.84) | -0.11(-1.81-1.59) |
| Blindness transient | 1 | 16.21(2.13-123.47) | 16.05(13.29) | 15.17(2.77-82.93) | 3.92(2.10-5.75) |
| Swelling face | 1 | 3.45(0.47-25.07) | 3.42(1.70) | 3.39(0.65-17.83) | 1.76(0.04-3.49) |
| Eyelids pruritus | 1 | 6.48(0.88-47.61) | 6.42(4.47) | 6.29(1.19-33.37) | 2.65(0.90-4.40) |
| Drug hypersensitivity | 1 | 3.86(0.53-28.12) | 3.83(2.07) | 3.79(0.72-19.96) | 1.92(0.20-3.65) |
| Instillation site irritation | 1 | 4.62(0.63-33.75) | 4.59(2.76) | 4.52(0.86-23.86) | 2.18(0.44-3.91) |
| Instillation site erythema | 1 | 5.51(0.75-40.36) | 5.46(3.58) | 5.37(1.02-28.42) | 2.43(0.68-4.17) |
| Product design issue | 1 | 259.53(16.11-4179.85) | 256.83(127.43) | 128.92(12.6-1318.96) | 7.01(4.64-9.38) |
| Treatment noncompliance | 1 | 28.83(3.62-229.78) | 28.54(23.92) | 25.78(4.54-146.44) | 4.69(2.79-6.59) |
| Intentional product use issue | 1 | 7.20(0.98-53.05) | 7.13(5.14) | 6.97(1.31-37.06) | 2.80(1.05-4.56) |

PT: preferred term; ROR: reporting odds ratio; CI: confidence interval; PRR: proportional reporting ratio; χ2: chi-squared; EBGM: empirical Bayesian geometric mean; IC: information component.
