# Supplementary material for: Safety signals of perfluorohexyloctane ophthalmic solution in patients with dry eye disease
Source: Front Med (Lausanne). 2026 May 28;13:1832619. doi: 10.3389/fmed.2026.1832619 (PMC13262190; doi:10.3389/fmed.2026.1832619)
Supplement: Supplementary file 8 [file Table_6.DOCX]

Supplementary Table 6 Risk signal of adverse reactions at the PT levels in people aged 65 and above

| PT | N | ROR(95%Cl) | PRR($\text{χ}^{\text{2}}$) | EBGM(95%Cl) | IC(95%Cl) |
| --- | --- | --- | --- | --- | --- |
| Product delivery mechanism issue | 16 | 22.90(13.27-39.51) | 21.66(268.05) | 18.51(11.73-29.22) | 4.21(2.51-5.91) |
| Eye irritation | 13 | 0.71(0.40-1.23) | 0.72(1.50) | 0.72(0.45-1.15) | -0.47(-2.15-1.21) |
| Vision blurred | 11 | 1.01(0.55-1.86) | 1.01(0.00) | 1.01(0.61-1.68) | 0.02(-1.66-1.69) |
| Inappropriate schedule of product administration | 11 | 13.61(7.22-25.65) | 13.12(111.43) | 11.93(7.02-20.27) | 3.58(1.88-5.27) |
| Product use issue | 10 | 14.49(7.44-28.18) | 14.01(108.52) | 12.65(7.25-22.08) | 3.66(1.96-5.36) |
| Intentional product use issue | 9 | 34.96(16.53-73.94) | 33.88(224.43) | 26.66(14.25-49.9) | 4.74(3-6.47) |
| Ocular hyperaemia | 7 | 1.07(0.51-2.28) | 1.07(0.04) | 1.07(0.57-2.01) | 0.10(-1.58-1.78) |
| Product use complaint | 7 | 11.98(5.47-26.27) | 11.71(62.65) | 10.76(5.58-20.76) | 3.43(1.72-5.13) |
| Accidental exposure to product | 7 | 4.28(1.99-9.17) | 4.20(16.56) | 4.09(2.16-7.74) | 2.03(0.35-3.72) |
| Exposure via skin contact | 7 | 78.58(30.24-204.22) | 76.66(319.62) | 47.24(21.25-105.04) | 5.56(3.76-7.36) |
| Product dose omission in error | 6 | 82.03(29.00-232.03) | 80.32(282.10) | 48.59(20.36-115.98) | 5.60(3.78-7.43) |
| Patient dissatisfaction with treatment | 6 | 73.83(26.65-204.54) | 72.28(263.74) | 45.55(19.42-106.86) | 5.51(3.69-7.32) |
| Wrong dose | 6 | 123.06(39.45-383.93) | 120.47(355.57) | 60.74(23.44-157.36) | 5.92(4.07-7.78) |
| Product packaging quantity issue | 6 | 9.96(4.30-23.07) | 9.77(43.78) | 9.11(4.51-18.4) | 3.19(1.48-4.89) |
| Visual impairment | 6 | 2.35(1.04-5.31) | 2.32(4.45) | 2.29(1.16-4.54) | 1.20(-0.49-2.88) |
| Foreign body sensation in eyes | 5 | 1.55(0.64-3.78) | 1.54(0.95) | 1.53(0.73-3.23) | 0.62(-1.06-2.30) |
| Eye pain | 4 | 0.44(0.16-1.19) | 0.45(2.75) | 0.45(0.2-1.04) | -1.14(-2.82-0.54) |
| Lacrimation increased | 4 | 1.00(0.37-2.69) | 1.00(0 .00) | 1.00(0.44-2.29) | 0.00(-1.68-1.68) |
| Therapy interrupted | 4 | 10.85(3.88-30.37) | 10.71(32.38) | 9.92(4.19-23.46) | 3.31(1.59-5.03) |
| Circumstance or information capable of leading to medication error | 4 | 37.59(12.18-115.99) | 37.07(107.40) | 28.58(11.13-73.38) | 4.84(3.03-6.64) |
| Cerebrovascular accident | 3 | 7.93(2.45-25.65) | 7.86(16.88) | 7.44(2.79-19.86) | 2.89(1.17-4.62) |
| Dry eye | 3 | 0.72(0.23-2.25) | 0.72(0.33) | 0.72(0.28-1.88) | -0.47(-2.15-1.21) |
| Product dose omission issue | 3 | 5.36(1.68-17.14) | 5.32(10.09) | 5.13(1.94-13.57) | 2.36(0.65-4.07) |
| Photophobia | 3 | 2.36(0.75-7.45) | 2.35(2.29) | 2.32(0.89-6.07) | 1.21(-0.48-2.90) |
| Burning sensation | 3 | 3.44(1.08-10.89) | 3.41(4.98) | 3.34(1.27-8.78) | 1.74(0.04-3.44) |
| Therapeutic product effect incomplete | 3 | 16.59(4.94-55.76) | 16.43(38.28) | 14.58(5.29-40.19) | 3.87(2.1-5.63) |
| Headache | 3 | 0.88(0.28-2.77) | 0.89(0.04) | 0.89(0.34-2.31) | -0.17(-1.85-1.51) |
| Product distribution issue | 3 | Inf(NaN-Inf) | Inf(361.45) | 121.47(0-Inf) | 6.92(4.71-9.13) |
| Product quality issue | 3 | 1.95(0.62-6.15) | 1.94(1.36) | 1.93(0.74-5.03) | 0.95(-0.74-2.63) |
| Product complaint | 3 | 12.17(3.69-40.10) | 12.05(27.65) | 11.04(4.07-29.96) | 3.47(1.72-5.21) |

Note: For "Product distribution issue" (n=3), ROR = Inf (NaN-Inf), PRR = Inf (361.45), EBGM = 112.47 (0-Inf), IC = 6.92 (4.19-9.13). This entry is presented descriptively only in this note, not included in the main table. Infinite values occur because this preferred term was not reported with other drugs, leading to mathematically undefined estimates. PT: preferred term; ROR: reporting odds ratio; CI: confidence interval; PRR: proportional reporting ratio; χ2: chi-squared; EBGM: empirical Bayesian geometric mean; IC: information component.
